# MicroRNAs in Rectal Cancer: Functional Significance and Promising Therapeutic Value

**DOI:** 10.3390/cancers12082040

**Published:** 2020-07-24

**Authors:** Laura Imedio, Ion Cristóbal, Jaime Rubio, Andrea Santos, Federico Rojo, Jesús García-Foncillas

**Affiliations:** 1 Cancer Unit for Research on Novel Therapeutic Targets, Oncohealth Institute, IIS- Fundación Jiménez Díaz-UAM, E-28040 Madrid, Spain; lauraimedio@gmail.com (L.I.); jaime.rubiop@quironsalud.es (J.R.); andreasantos.asc@gmail.com (A.S.); 2 Translational Oncology Division, Oncohealth Institute, IIS- Fundación Jiménez Díaz-Universidad Autonoma de Madrid (UAM), E-28040 Madrid, Spain; 3 Medical Oncology Department, University Hospital “Fundación Jiménez Díaz”, UAM, E-28040 Madrid, Spain; 4 Pathology Department, IIS- Fundación Jiménez Díaz-UAM, E-28040 Madrid, Spain; Frojo@fjd.es

**Keywords:** microRNA, signaling, therapy, rectal cancer

## Abstract

It is well-known that microRNAs (miRNAs) are critical mediators of initiation and disease progression in many human cancers. Rectal cancer is a highly prevalent tumor, accounting for around one third of newly diagnosed colorectal cancers. The usefulness of miRNAs as clinical biomarkers predictive of the outcome and response to chemoradiotherapy has been well-reported for rectal cancer. However, the existing literature on their functional and therapeutic impact needs to be put in context to clarify their role in disease pathogenesis. Therfore, this review is focused on the functional relevance of miRNAs as key regulators of signaling pathways in rectal cancer and their potential therapeutic value as novel molecular targets in this disease.

## 1. Introduction

Colorectal cancer (CRC) is the third most commonly diagnosed malignancy and the second leading cause of cancer-related deaths in the world, with approximately 1.8 million new cases and 900,000 deaths annually [1]. Rectal carcinoma represents approximately 30% of all colorectal tumors and is defined as tumors arising within 15 cm of the anal verge [2]. Both environmental and genetic factors play a major role in the pathogenesis of rectal cancer [3]. Male sex and increasing age have been associated with higher incidence rates [4]. Rectal cancer presents several risk factors characterizing westernized lifestyles, such as obesity, sedentary lifestyles, poor diets, an excessive alcohol intake, and smoking [5,6]. Regarding mortality, metastasis and recurrence are the main causes of death [5]. Declining rates are only seen in highly developed countries, primarily attributed to advances in cancer treatment and management [7,8]. Due to the anatomical localization of the rectum in the narrow pelvis surrounded by the bladder, internal genital organs, nerves, and large vessels, it poses a challenge to the clinical approach in rectal carcinoma. Moreover, it also differs from colon cancers in its metastatic pattern, with the lungs being the first organs to present metastasis in rectal adenocarcinoma rather than the liver, as occurs in colon cancer. Therefore, the clinical management of rectal cancer involves different surgical approaches and therapeutic strategies [9]. Most patients diagnosed in the early stages of rectal cancer can be managed with surgery alone. However, onset of the disease is occult and the best time for treatment is lost [5]. A significant proportion of patients with locally advanced disease (LARC) will potentially benefit from downsizing or downstaging tumors with a clinical stage T3 or T4 prior to total mesorectal excision (TME). Therefore, neoadjuvant 5-fluorouracil (5-FU)-based chemoradiotherapy represents the current standard treatment in this subgroup of patients [2]. The response to chemoradiotherapy (CRT) varies widely, from complete responses to complete resistance. In 15–20% of cases, a complete response to radiotherapy is observed and patients can potentially avoid surgery and associated morbidities [7,10]. While these results are promising, there are a significant number of patients that will not benefit from CRT and will endure the side effects secondary to radiation exposure, including an increased risk of anorectal and genitourinary dysfunction, which negatively impacts their quality of life, and excessive tissue oedema, leading to the cancellation or postponement of surgical plans [2]. Notably, there is a current lack of well-established biomarkers for predicting responses to neoadjuvant CRT in LARC patients and all patients receive the same therapy [11]. Moreover, it is necessary to further understand the molecular mechanisms that govern rectal cancer progression, in order to develop alternative therapeutic strategies in non-responder cases. Currently, both the identification of novel biomarkers and the development of alternative therapeutic strategies to impair disease progression are being pursued [12]. In this regard, the potential clinical value of microRNAs (miRNAs) has progressively emerged in the last decade [13].

MiRNAs are small (18–25 nucleotides) single-stranded and non-coding RNAs that downregulate gene expression at the post-transcriptional level through binding to target mRNAs and triggering their degradation or translational blocking [14]. They play a key role in the regulation of biological processes, such as apoptosis, cell differentiation, development, and proliferation, and it is believed that up to 30% of human genes are regulated by miRNAs [15,16]. miR-mRNA binding is specific due to the sequence complementarity of the “seed” region (7–8 nucleotides at the 5′ end) of the miRNA with the 3′ untranslated region (3′ UTR) of mRNAs. A single miRNA can target hundreds of different mRNA targets, while an individual mRNA can be simultaneously regulated by multiple different miRNAs [11,17]. Most miRNAs are located in intergenic regions and their biogenesis involves several steps. First of all, miRNA genes are transcribed by RNA polymerase II in the nucleus generating pri-miRNAs, which are capped and polyadenylated. These primary transcripts include hundreds of nucleotides and form hairpin loops. The microprocessor complex, which includes nuclear RNase III DROSHA and a double-stranded RNA-binding protein DGCR8, cleaves the primary structure of the pri-miRNAs. The products of this reaction are pre-miRNAs (60–70 nucleotides), which are transported to the cytoplasm by exportin-5 and later processed by DICER, which is a cytoplasmic RNase III enzyme. The guide strand of the resulting 18-25 nucleotide-long mature miRNA is incorporated into the miR-induced silencing complex (RISC), which contains Argonaute family proteins, while the passenger strand is removed [15,16,17]. RISC migrates to processing bodies (P-bodies) and delivers miRNA to bind sequence complementary mRNA, in order to mediate gene silencing by targeted mRNA degradation and translational repression [10,11].

Given the great impact of miRNAs on gene expression, it is not surprising that miRNA deregulation contributes to the initiation, progression, and dissemination of any type of human tumor [10,18]. They can act as oncogenic miRNAs (oncomiRNAs) or tumor suppressor miRNAs, depending on the function of the targeted mRNA [13]. Both the overexpression of specific oncomiRNAs and silencing of tumor suppressor miRNAs have been associated with the tumorigenesis of rectal cancer by inhibiting key components of the main signaling pathways altered in this disease. The overexpression of a miRNA can be due to the amplification of its coding gene or augmented transcription, while miRNA downregulation can be caused by epigenetic silencing, deletion of its coding gene, or defective biogenesis [15]. MiRNAs can be secreted into bodily fluids with minimal degradation, present a high stability during storage, and can be easily quantified using real-time quantitative polymerase chain reaction (RT-qPCR) microarray, bead array, or sequencing approaches [14,19]. All of these advantages facilitate their use in the clinical setting and miRNAs are emerging as stable and non-invasive biomarkers regarding diagnosis, staging, and prognosis in rectal cancer management [11,15]. Although several miRNAs have already been described as biomarkers predictive of responses to CRT in rectal cancer patients [20], there are a lack of robust established markers available in the clinical routine [21]. In recent years, changes in miRNA expression patterns and their functional roles in rectal pathogenesis have been increasingly studied [10]. Moreover, they are emerging as promising candidates for targeted therapies since their function can be ectopically manipulated [13,15].

## 2. MiRNAs as Regulators of Signaling Pathways in Rectal Cancer

Rectal tumor cells present complex alterations in their genome, transcriptome, and epigenome that affect key signaling pathways involved in tumor initiation and progression of the disease. These signaling cascades include phosphatidylinositol-3-kinase (PI3K); the epidermal growth factor receptor (EGFR); the Wnt/β-catenin, IGFR, TGFβ, p53, and DNA mismatch-repair pathways; extracellular matrix (ECM) regulators; and epithelial to mesenchymal transition (EMT) transcription factors [15,19,22]. Some components of these pathways are frequently inactivated, such as PTEN, APC, TP53, and SMAD4, while others are constitutively activated (K-Ras) or overexpressed (Myc) [13]. Recently, evidence has shown that miRNAs also contribute to the dysregulation of these signaling pathways by targeting several genes and representing a complex regulatory system of central biological importance [23]. Therefore, analyses of these miRNAs are crucial for exploring the tumorigenesis mechanism of rectal cancer and identifying novel relevant therapeutic targets in this disease [15].

The first miRNA profiling was performed in colorectal cancer samples by Cummins et al. in 2006 [24]. This analysis showed that miRNAs were aberrantly expressed in CRC tissues and cell lines compared to the normal counterparts and provided the first evidence of miRNAs’ role in tumorigenesis [25,26]. Since several studies have shown that colon and rectal carcinomas differ in their global miRNA profiles and LARC requires different treatment strategies, it is important to study them separately [20]. Whilst several miRNA expression signatures for CRC have been performed to date, only limited data on miRNAs in rectal cancer are currently available [27].

The rectal cancer miRNAome was described by Gaedcke et al. Forty nine miRNAs were differentially expressed, but only thirteen of them were rectal cancer specific and have not previously been reported in colon cancer (miR-492, miR-542-5p, miR-584, miR-483-5p, miR-144, miR-2110, miR-652, miR-375, miR-147b, miR-148a, miR-190, miR-26a/b, and miR-338-3p) [19]. Another study focused on rectal cancer was performed by Li and colleges, in which twenty four novel aberrantly expressed miRNAs were identified, including miR-31, miR-126 miR-18a, miR-135b, miR-21, miR-145, and miR-143 [28]. Understanding their functional effects and underlying molecular mechanisms is required to improve our knowledge of the disease pathogenesis and develop alternative therapeutic strategies [27,29]. This review will be focused on specific miRNAs that modulate the signaling pathways critical for rectal cancer (Table 1).

### 2.1. PI3K/AKT/PTEN Pathway

The activation of phosphatidylinositol-3-kinase (PI3K)/Akt signaling plays a vital role in development and progression in a broad range of cancers presenting alterations in any step of the pathway [30]. In rectal cancer, advanced pathway analysis has revealed that this route is the most affected in the sporadic late-onset subgroup [22], with all of the PI3K/Akt pathway components found to be either mutated or overexpressed [30]. Johnson et al. revealed that targeting these components could effectively inhibit tumor growth and metastatic capability, increase cell apoptosis, and sensitize CRC cells to chemotherapy [44]. Furthermore, several miRNAs found to be deregulated in rectal cancer have been shown to target PI3K/Akt components.

Meltzer et al. studied miR-141-3p and miR-375—two exosomal miRNAs that were elevated in blood samples of LARC patients [30]. MiR-141-3p was reported to target the phosphatase and tensin homolog (PTEN), which is an important regulatory component of the PI3K/Akt pathway [33]. The inhibition of this tumor suppressor gene results in the activation of the signaling pathway. Regarding miR-375, Biton and colleagues showed that PTEN inhibition and subsequent PI3K-Akt activation resulted in increased miR-375 expression levels [31]. Moreover, miR-375 upregulation has been found to enhance the immunosuppressive environment in the tumor [45]. Therefore, it was postulated that high miR-141-3p levels upregulate the PI3K-Akt pathway and increase miR-375 expression, which subsequently promotes an immunosuppressive environment for tumor cells [30].

Another miRNA whose expression is dysregulated in rectal cancer is miR-126. Guo and colleagues found that miR-126 can deregulate the PI3K pathway by targeting p85β in CRC cells, which is involved in stabilizing and propagating the PI3K signal and its downstream signaling effector AKT [32]. It was suggested that miR-126-dependent p85b inhibition provides a negative regulation of this signaling pathway, thereby affecting cell proliferation and the colony forming ability [25].

Among the oncomiRs, mir-21 is the most frequently upregulated miRNA in CRC and presents an important function in the progression of the disease [32]. Drebber et al. discovered that PTEN is strongly repressed by miR-21 in CRC tissues, leading to PI3K signaling activation that induces tumor growth and metastatic progression [33].

### 2.2. EGFR Pathway

The epidermal growth factor receptor (EGFR) pathway contributes to the proliferation, differentiation, and development of a broad spectrum of solid tumors, including rectal cancer. EGFR overexpression leads to the initiation of a cascade of downstream effectors that mediate tumor growth, survival, angiogenesis, and metastasis [15,25]. Ras is a guanine nucleotide-binding protein situated downstream of EGFR that presents three different isoforms: H-Ras, K-Ras, and N-Ras [25]. K-Ras is one of the most frequently altered oncogenes in CRC, mainly through mutations that lead to constitutive activation of the protein [44]. Moreover, the overexpression of Ras is also achieved as a result of the dysregulation of different miRNAs.

Therefore, let-7 and miR-143 are associated with Ras regulation in cancer cells, subsequently altering cell proliferation, cell adhesion, and apoptosis [25]. Regarding the let-7 family of miRNAs (let-7a-h), it is specifically downregulated in many cancer types, including rectal cancer, and targets both Ras and Myc oncogenes [19]. MiR-143 has been described to be downregulated in tumor biopsies of LARC patients [19], functioning as a tumor suppressor miRNA [33,46]. In addition to inhibiting Ras expression, miR-143 has also been demonstrated to reduce the phosphorylation of ERK1/2—a downstream effector of the EGFR pathway—in LoVo cells [15,25,46].

### 2.3. Wnt/β-Catenin Pathway

The Wnt signaling pathway is known to regulate a wide range of critical cellular functions via the regulation of β-catenin expression levels. This protein is a crucial growth stimulatory factor that plays a vital role in cell proliferation, invasion, differentiation, and other signaling pathways through the activation of transcription factors such as c-Myc and cyclin D [35]. Inactivation of the adenomatous polyposis coli (APC) gene—a tumor suppressor—is a major initiating event in CRC tumorigenesis and leads to overactivation of the Wnt pathway due to β-catenin release from the APC, glycogen synthase, and the axin degradation complex [25]. This results in dysregulated cell proliferation and tumor development [1]. Several miRNAs have been found to regulate this pathway in rectal cancer.

In 2019, Shao et al. studied the role of miR-101-3p—a rectal cancer downregulated miRNA—in HT29 and SW620 cell lines [35]. Firstly, they identified miR-101-3p as an inhibitory target of the small nucleolar RNA host gene 6 (SNHG6), which is an overexpressed lncRNA and associated with carcinogenesis in several malignancies [47,48,49]. It has been reported to promote carcinogenesis by acting as an miR-101-3p sponge in CRC. This study identified SNHG6 as an oncogenic lncRNA that promotes CRC progression via regulating miR-101-3p expression and Wnt/β-catenin signaling [35].

MiRNAs also represent a novel mechanism for APC regulation in rectal cancer. Nagel and colleges showed that miR-135a and miR-135b directly target the APC 3-untranslated region (3′ UTR), suppressing its expression and activating Wnt signaling [36]. Both miR-135a/b were found to be upregulated in colorectal cancer and correlated with low APC levels. Therefore, an alteration in the miR-135 family might be one of the early events in the pathogenesis of rectal cancer [15].

### 2.4. IGFR Pathway

Type 1 insulin-like growth factor receptor (IGF-IR) and insulin receptor substrate-1 (IRS-1) are important cell regulators whose activation triggers a strong mitogenic, antiapoptotic, and anti-differentiation stimulus [25]. IRS-1 is the IGF-IR physiological co-activator and its expression levels are often increased in several human cancers [15]. Moreover, upregulated IGF1 has been found to be correlated with an increased risk of CRC [27].

MiR-145 is a tumor suppressor miRNA frequently downregulated in rectal cancer, whose role in the IGFR pathway has been demonstrated in colon cancer cells [15]. Shi and colleagues reported that miR-145 directly targets IRS-1 and dramatically inhibits the growth of colon cancer cells [37]. La Rocca et al. demonstrated that IGF-IR is another miR-145 target [38]. Since IGF-IR and IRS-1 are both regulated by miR-145, its downregulation could contribute to disbalance between proliferation and differentiation in rectal cancer cells via the insulin signaling pathway [25].

MiR-195 has been reported to be deregulated in rectal cancer among many other malignant tumors, including non-small cell lung, cervical, and breast cancer [19]. In 2019, Wang et al. confirmed that IGF1 is an effective target of this miRNA [27]. MiR-195 expression negatively correlated with the cell survival rate and migratory and invasive capacities of rectal tumor cells. The tumor suppressive ability of miR-195 was dependent on IGF1 inhibition [27].

### 2.5. Other Signaling Pathways

A study by Tong and co-workers showed that miR-125a-5p, which is a tumor suppressor miRNA downregulated in rectal cancer [28,39], was negatively associated with cell proliferation in CRC cell lines. Three antiapoptotic genes—BCL2, BCL2L12, and Mcl-1—were identified as miR-125a-5p targets [39]. Therefore, low miR-125a-5p expression levels quantified in rectal tumor samples might promote antiapoptotic signals, as observed in colon cancer cells.

Another miRNA related to antiapoptotic signaling is miR-21 [10]. This miRNA contributes to the regulation of cell death in rectal cancer by modulating PDCDR4 expression [50]. PDCDR4 is a programmed cell-death protein that regulates intravasation through the invasion-related urokinase receptor gene (uPAR), and induces apoptosis through the inhibition of 12-O-tetradecanoylphorbol 13-acetate (TPA)-dependent tumor transformation and progression [15,33]. Moreover, miR-21-induced PDCDR4 downregulation has been shown to be associated with cell invasion and metastatic potential in CRC patients [25,50].

Furthermore, miR-21 is known to regulate multiple genes associated with cellular motility and extracellular matrix (ECM) remodeling, which has crucial roles in tumor growth, survival, invasiveness, and metastasis [10,33]. Matrix metalloproteinases (MMPs), as well as the urokinase plasminogen activator (uPA) cascade components, represent key factors in this process. Gabriely et al. discovered a negative regulation of miR-21 on RECK and TIMP3 genes, which are inhibitors of MMPs [40]. Although these observations were obtained from a glioblastoma model, the upregulation of miR-21 in rectal cells has been shown to increase migration and invasion abilities and might produce similar results [15].

Finally, a study performed by Lopes-Ramos et al. described SATB1 as a target of miR-21-5p [41]. MiR-21 plays a role in microvillus-like formation in CRC cell lines by targeting Sprouty2 (SPRY2), which is a protein directly involved in branching morphogenesis and neurite outgrowth inhibition [42]. The work by Wang et al. reported that miR-21 inhibits cdc25a, leading to cell cycle arrest at the G1/S checkpoint and the subsequent inhibition of colon cancer tumor growth [43].

## 3. MiRNAs as Potential Therapeutic Targets in Rectal Cancer

The standard treatment for LARC patients is 5-FU-based neoadjuvant CRT [51]. However, a complete pathologic response is only observed in approximately 20% of cases, with about 40% of patients presenting a poor or absent pathological response after CRT [52,53]. Therefore, it remains urgent to elucidate the complex mechanism of CRT resistance in rectal cancer and identify potential molecular targets to develop novel effective treatments [54,55]. The existence of therapy-resistant cancer stem cells (CSCs) offers a potential explanation for cancer treatment failure. Moreover, a strong relationship between the CSC phenotype, epithelial-mesenchymal transition (EMT) pathways, and cell survival has been described. Notably, the targeting of these pathways to enhance CRT sensitivity in LARC patients has been proposed in the literature [56].

Nowadays, the rapidly expanding knowledge on aberrant patterns of miRNA expression, along with functional analyses of specific signaling pathways, has exposed the remarkable potential of manipulating miRNA levels, which may provide a new therapeutic strategy in LARC [26]. The advantage of using miRNAs over traditional anticancer drugs is that miRNAs mediate potent and specific gene silencing and to date, no cancer resistance has been reported in terms of treatment targeting miRNAs, making them attractive therapeutic targets [18,57]. However, since a specific miRNA is able to target several hundreds of genes, it may be difficult to determine the global carcinogenic effect of that miRNA and to elucidate the effects and toxicity profiles that may be associated with its therapeutic application [58].

Several techniques have been progressively developed to facilitate the clinical application of miRNA therapy. The chosen strategy depends on whether miRNA expression needs to be downregulated, including antisense oligonucleotide (AMO) or antagomirs, locked nucleic acids (LNA), peptide nucleic acid (PNA), or the newest miRNA sponge and miRNA masking techniques, or restored, by miRNA mimics or viral vector-encoded miRNA replacement [26,59]. They can be easily administered through local or parenteral injections and are sufficiently taken up by the tissue, without the need to develop formulations. Despite all of these advantages, miRNA therapy for rectal cancer has still not reached clinical trials and only a few miRNAs altered in CRC have been studied in vivo. In addition, potential undesirable side effects have to be considered after miRNA modulation due to both the expression and function of certain miRNAs being tissue-specific or different between distinct tissues [60,61,62].

Although more than 70 miRNAs have been described as biomarkers predictive of responses to CRT in LARC patients [20], only a limited number of studies have demonstrated the potential therapeutic value of certain miRNAs as novel molecular targets in rectal cancer [63], which have been summarized in Table 2.

### 3.1. MiRNAs as Potential Therapeutic Targets in 5-Fluorouracil-Based Chemoresistance

5-FU is a uracil analog that inhibits thymidylate synthase, which is the core enzyme in pyrimidine nucleotide synthesis, blocking the synthesis of DNA and disrupting RNA processing [66]. Resistance to 5-FU is a major obstacle to successful treatment and identifying its underlying molecular mechanisms is one of the major challenges for creating new opportunities for LARC treatment. Nowadays, there is evidence of miRNAs being involved in 5-FU resistance or sensitivity [71]. Moreover, cancer stem cells (CSCs) have been postulated to play an important role in chemoresistance [64]. CSCs in CRCs are generally characterized by the expression of CD133+ and a self-renewing capacity [64].

Of note, Jin et al. studied a miRNA downregulated in rectal tissues that has been previously shown to be involved in chemoresistance to thymidylate synthetase inhibitors in LARC patients [64,72]. They found that miR-450b-5p expression was significantly inhibited after 5-FU treatment in CRC cells. Moreover, its overexpression in 5-FU-resistant HT-29 cells led to a decreased cell viability and enhanced apoptosis. SOX2, an essential factor in stem cells, was identified as a direct miR-450b-5p target, and the expression of this miRNA negatively correlated with CRC CD133+ cells. In summary, Jin and co-workers demonstrated that miR-450b-5p inhibits the stemness and development of 5-FU chemoresistance in CRC cells and may represent a novel therapeutic target for improving chemotherapy in LARC [64].

Another work showed that the restoration of miR-577 expression significantly suppressed proliferation, colony formation, and in vivo tumor growth, and induced G0/G1 cell cycle arrest in CRC cells. Moreover, its overexpression in 5-FU-resistant SW480 cells enhanced the sensitivity to the chemotherapeutic agent. Mechanistically, the authors identified that these effects were mediated by the direct target of heat shock protein 27 (HSP27). Therefore, these findings suggest that miR-577 may have potential therapeutic value in LARC [65].

Recently, Xu et al. found that miR-375-3p is downregulated in CRC cell lines and tissues and directly targets thymidylate synthase (TS), which is also a known 5-FU target. In order to co-transport 5-FU and miR-375-3p into cells, lipid-coated calcium carbonate nanoparticles (NPs) were designed. The advantages are their excellent biocompatibility and degradability, efficient and rapid release of drugs in a weakly acidic tumor microenvironment, improved therapeutic efficacy, and prolonged circulation time. The restoration of miR-375-3p levels enhanced 5-FU sensitivity and induced cell apoptosis and cell cycle arrest, inhibiting cell growth, migration, and invasion in vitro. Therefore, targeting miR-375-3p seems to be an effective method for modulating and enhancing 5-FU antitumor effects [66].

Liu and colleagues analyzed miR-139-5p—a miRNA under-expressed in 5-FU-resistant CRC cell lines and rectal tumors after CRT. MiR-139-5p inhibited NOTCH-1 expression and its downstream molecules MRP-1 and BCL-2, increasing cell apoptosis and the sensitivity to 5-FU. Moreover, miR-139-5p impaired cell migration and invasion. These data suggest that the miR-139-5p/NOTCH-1 signaling axis might be a promising therapeutic target for chemoresistance in rectal cancer [67].

Lastly, our group has recently reported that miR-199b is a frequently downregulated tumor suppressor miRNA in LARC that directly targets the PP2A inhibitor SET Nuclear Proto-Oncogene (SET), which has been previously shown to be involved in 5-FU resistance. Our findings demonstrated that miR-199b enhances the sensitivity of SW480 and HT-29 cell lines to 5-FU, presenting cell growth reduction and increased caspase-dependent apoptosis when ectopically overexpressed. Moreover, the restoration of miR-199b levels in acquired 5-FU-resistant models re-sensitized cells to this drug. Altogether, these results highlight the potential impact of the miR-199b/SET axis as a novel druggable target in LARC [21].

### 3.2. MiRNAs as Potential Therapeutic Targets in Radioresistance

Radioresistance represents a major clinical challenge in the treatment of advanced rectal cancer [68]. Importantly, increasing evidence in the literature suggests the involvement of miRNAs in the cellular response to ionizing radiation.

CSCs have gained interest as novel therapeutic targets for overcoming resistance to traditional anticancer therapy, such as radiation. A number of studies have correlated the acquisition of the EMT phenotype with enrichment of the CSC population, leading to a therapeutic resistant phenotype. Ha Thi and colleagues, using small RNA sequencing in rectal cancer cell lines, selected miR-130a as a promising candidate, since it was dramatically upregulated in radiosensitive cells. Moreover, a forced expression of miR-130a suppressed cell growth and colony formation upon irradiation treatment. Mechanistically, they found that this miRNA reversed the IR-induced EMT phenotype and inhibited invasion and DNA damage repair by directly targeting SOX4. It is a potential oncogene whose expression correlated with increased levels of EMT markers and the enhancement of β-catenin/TCF activity. Both in vitro and in vivo models showed a dramatic increase of radiosensitivity and therefore, miR-130a was selected as a potential radiosensitizer in rectal cancer [68].

The work by Zhu et al. analyzed SNAI1—an EMT mediator and member of the transcriptional repressor SNAIL family [56]. Moreover, SNAI1 repressed miR-145 promoter activity and subsequently its expression. This miRNA has been shown to target multiple mediators of EMT and stem cell transcription factors, such as OCT4, SOX2, and KLF4. These novel findings suggest that the SNAI1/miR-145 axis represents a novel therapeutic target for overcome radioresistance in LARC [56]. Additionally, miR-145 has already been complexed with polyethylenimine as a vehicle to target cancer xenografts [73].

Using different approaches, several studies have performed miRNA expression profiling analyses in order to identify miRNAs that serve as molecular targets to sensitize IR-resistant cancer cells [55]. The advantage of this technique is the large number of candidate markers that can be obtained simultaneously [74]. Salendo et al. identified thirty-six miRNAs that significantly correlate with the sensitivity to CRT. The functional relevance of selected miRNAs (let-7g, miR-132, miR-224, and miR-320a) was established by transfecting the respective mimics into SW480 and SW837 cells and all of them induced a shift of IR-sensitivity. Therefore, targeting these miRNAs with oligonucleotides represents a potentially useful therapy that needs to be investigated further [55].

Radiation-induced rectal injury is a major side-effect observed in patients caused by radiotherapy, which damages the adjacent normal tissues in rectal cancer patients. Ge et al. have recently reported that miR-122-5p is upregulated in both patient serum and mice rectal tissues after irradiation. The authors postulated a role of this miRNA in radioresistance and radiation-induced rectal injury and found that CCAR1, which is a transcriptional coactivator of Wnt/β-catenin signaling, was a direct target of miR-122-5p. When, in combination with IR, this miRNA was overexpressed or CCAR1 was silenced in HIEC cells, inhibited cell survival and increased cell apoptosis were observed in vitro as a result of the re-sensitization of these cells to radiation. Moreover, an in vivo injection of miR-122-5p inhibitor significantly reduced rectal injury subsequent to radiation in mice. These results suggest that miR-122-5p improves the radiosensitivity in HIEC cells through targeting CCAR1 and aggravates radiation-induced rectal injury [69].

### 3.3. MiRNAs as Potential Therapeutic Targets in Combination with Chemoradiotherapy

MiRNAs can also be used in combination with standard CRT to target additional signaling pathways involved in rectal cancer progression. This approach could help to downsize the tumor in non-responder LARC patients.

In 2019, Wang et al. investigated whether miR-195 could inhibit rectal cancer progression, since it was found to be more downregulated in rectal cancer cells than in epithelial cells from the rectal mucosa. Of note, ectopic miR-195 expression significantly decreased the cell viability, enhanced apoptosis, and reduced migratory and invasive abilities in a IGF1-dependent manner, and also blocked the PI3K/AKT pathway. These results highlight the potential therapeutic value of miR-195 in rectal cancer [27].

Another miRNA widely studied in rectal cancer is miR-31, which is overexpressed in several tumors, including liver, lung, and ovarian cancer. Mu et al. found significantly higher miR-31 expression in rectal cancer tissues compared to the normal counterparts. Functionally, miR-31 overexpression increased proliferation and invasiveness, and these effects were confirmed by performing mirror experiments [5].

MiRNA-129-5p plays a role in proliferation and invasion and has been proposed as a novel therapeutic target in LARC. This miRNA is downregulated in rectal cancer tissues and its restoration leads to inhibited proliferation, invasion, and migration, and enhanced apoptosis. In vivo experiments showed an inhibition of tumor growth after miR-129-5p upregulation. E2F7 was identified as an miR-129-5p target that mediates miR-129-5p-induced effects. These results suggest that miR-129-5p could serve as an effective target for LARC that should be further studied [70].

Three more miRNAs have been demonstrated to play roles in the proliferation, migration, and apoptosis of rectal cancer cells. First, miRNA-144 is downregulated in rectal cancer and its restoration suppressed cell viability, migration, and proliferation by targeting the oncogene ROCK1, which is involved in reorganization of the actin cytoskeleton during motion. Therefore, this tumor suppressor miR-144 has been proposed as a novel therapeutic target for rectal cancer treatment [29]. The second one is miR-381, another altered tumor suppressor in rectal cancer that directly inhibits UBE2C expression. The function of this protein takes place in the degradation of APC substrates that control the cell cycle and it was found to be overexpressed in rectal carcinoma samples. This oncogene has been shown to promote cell proliferation and invasion and inhibit cell apoptosis after miR-381 overexpression both in vivo and in vitro. This suggests the potential usefulness of miR-381 as a therapeutic target in rectal cancer [51].

Third, miR-19a-3p is downregulated in rectal cancer cells and tissues. Su et al. studied its functional significance by modulating its expression in CRC cells, observing that miR-19a-3p impairs cell proliferation, migration, and invasion, while its inhibition shows the opposite effects. Moreover, this study elucidated that miR-19-3p induced cell apoptosis through promoting reactive oxygen species (ROS) accumulation as a result of FAS targeting [57].

## 4. Conclusions

Despite many efforts to confront rectal cancer, it remains one of the main causes of cancer-related deaths, primarily due to metastasis and recurrence [5]. Since the mainstay of treatment for locally advanced rectal cancer heavily relies on neoadjuvant 5-FU-based CRT, resistance to CRT is still a major challenge [51,71]. To date, significant advances in the use of miRNAs as therapeutic targets have been made. However, several limitations have to be considered before miRNA-based therapies become a reality. First, it is critical to validate the multiple mRNA targets of each miRNA and to elucidate the complex pathways in which they take part, in order to predict the side effects and toxicity profiles that may be associated with miR-based therapeutics [26]. Secondly, in vivo investigations followed by early phase clinical studies are essential for identifying the real impact of miRNA therapy. Moreover, developing more efficient delivery systems that are site-specific, in order to minimize the exposure of healthy cells to these therapies, and that promote a good uptake of miRNAs into the target tissue, is critical [26]. Despite this, there is a promising future in the use of miRNA and further investigations must be conducted.

## Figures and Tables

**Table 1:** Altered microRNAs (miRNAs) regulating key signaling pathways in rectal cancer.

Pathway	MiRNA	Role	Putative Target	References
PI3K/Akt	miR-141-3p	Oncogenic	PTEN	[30]
	miR-375	Oncogenic	Klf5	[31]
	miR-126	Oncogenic	p85β	[32]
	miR-21	Oncogenic	PTEN	[33]
EGFR	let-7 family	Tumor suppressor	Ras and Myc	[34]
	mir-143	Tumor suppressor	Ras	[33]
Wnt	miR-101-3p	Tumor suppressor	β-catenin and TCF4	[35]
	miR-135a/b	Oncogenic	APC	[36]
IGFR	miR-145	Tumor suppressor	IRS-1	[37]
			IGF-IR	[38]
	miR-195	Tumor suppressor	IGF1	[27]
Other signaling pathways	miR-125a-5p	Tumor suppressor	BCL2, BCL2L12 and Mcl-1	[39]
	miR-21	Oncogenic	RECK and TIMP3	[40]
			SATB1	[41]
			SPRY2	[42]
			cdc25a	[43]

**Table 2:** MiRNAs as potential therapeutic targets in rectal cancer.

Therapeutic Impact	MiRNA	Target	Effect	References
Chemotherapy	miR-450b-5p	SOX2	Reduced cell viability and elevated DNA fragmentation levels and caspase-3 activity.	[64]
	miR-577	HSP27	Inhibited cell proliferation and colony formation.	[65]
	miR-375-3p	TYMS	Induced cell apoptosis and cell cycle arrest and inhibited cell growth, migration, and invasion.	[66]
	miR-139-5p	NOTCH-1	Increased cell apoptosis and inhibited cell migration and invasion.	[67]
Radiotherapy	miR-130a	SOX4	Suppressed cell growth and colony formation, reversed IR-induced EMT phenotype, and inhibited invasion and DNA damage repair.	[68]
	miR-145	OCT4, SOX2 and KLF4	Inhibited EM transition and stem cell phenotype.	[56]
	miR-122-5p	CCAR1	Sensitized HIEC cells to radiation, inhibited cell survival, and increased cell apoptosis, but increased radiation-induced rectal injury.	[69]
Others	miR-195	IGF1	Overexpression decreased cell viability, enhanced apoptosis, and reduced migratory and invasive capacities.	[27]
	miR-31		Increased proliferation and invasiveness.	[5]
	miR-129-5p	E2F7	Inhibited proliferation, invasion, and migration, while the apoptosis ability was enhanced. Inhibition of tumor growth.	[70]
	miR-144	ROCK1	Suppressed cell viability, migration, and proliferation.	[29]
	miR-381	UBE2C	Inhibited cell proliferation, invasion, and cell apoptosis.	[51]
	miR-19a-3p	FAS	Induced cell apoptosis through ROS accumulation. Reduced cell proliferation, migration, and invasion.	[57]
